# The effect of cell penetrating peptide-conjugated coactivator-associated arginine methyltransferase 1 (CPP-CARM1) on the cloned mouse embryonic development

**DOI:** 10.1038/s41598-018-35077-0

**Published:** 2018-11-13

**Authors:** Jae-Il Bang, Eun-Hye Lee, Ah Reum Lee, Jin Il Lee, Seo Hye Choi, Dong-Won Seol, Chang-Hwan Park, Dong Ryul Lee

**Affiliations:** 1 Department of Biomedical Science, CHA University, Seongnam, 13488 Korea; 2 Graduate School of Biomedical Science and Engineering, Hanyang University, Seoul, 04763 Korea; 3 Fertility Center of CHA Gangnam Medical Center, College of Medicine, CHA University, Seoul, 06135 Korea; 4 CHA Stem Cell Institute, CHA University, Seongnam, 13488 Korea

## Abstract

Abnormalities in gene expression that negatively affect embryonic development are frequently observed in cloned embryos generated by somatic cell nuclear transfer (SCNT). In the present study, we successfully produced a cell-penetrating peptide (CPP)-conjugated with coactivator-associated arginine methyltransferase 1 (CARM1) protein from mammalian cells and confirmed introduction into donor somatic cells and cloned 8-cell embryos within 3 hours after addition to culture medium. In addition, H3R17 dimethylation and embryonic development up to the blastocyst stage were increased in the group treated with exogenous CPP-CARM1 protein compared with the untreated group (control). Interestingly, the number of total cells and trophectoderm in blastocysts as well as implantation rate were significantly increased in the CPP-CARM1 protein-treated group. However, the cell number of inner cell mass (ICM) was not changed compared with the control group; similarly, expression of pluripotency-related genes *Oct4 and Nanog* (ICM markers) was not significantly different between groups. On the other hand, expression of the implantation-related gene *Cdx2* (trophectoderm marker) was transiently increased after treatment with CPP-CARM1 protein. On the basis of these results, we conclude that supplementation with exogenous CPP-CARM1 protein improves embryonic development of cloned embryos through regulation of histone methylation and gene expression. In addition, our results suggest that CPP-CARM1 protein may be a useful tool for strengthening implantation of mammalian embryos.

## Introduction

Somatic cell nuclear transfer (SCNT) is the process by which the cytoplasm of a recipient oocyte reprograms a nucleus from a differentiated somatic donor cell, resulting in the production of cloned embryos with the genetic information of the donor cell. This technique was first described for the embryonic development of *Xenopus* in a report by Gurdon^1^. Subsequent research has confirmed that fully differentiated somatic cells are capable of cell-fate switching through reprogramming^1^. Since the study of Gurdon, SCNT technology has successfully produced animals from cloned embryos of various species of mammals^2–4^. However, poor quality of developmental patterns attributable to reduced cell number and altered gene expression were frequently observed in most cloned embryos compared with *in vivo*- and *in vitro*-fertilized embryos^5^. To overcome this problem, researchers have sought to produce improved cloned embryos through supplementation of the culture medium with chemical reagents^6–8^ and growth factors^9,10^.

A number of epigenetic strategies for manipulating embryonic development without changing gene sequences have recently been investigated^11–13^, some of which have been applied to the production of cloned embryos^14,15^. Previous studies reported that application of the histone modifying enzymes, lysine (K)-specific demethylase 4D (KDM4D) to cloned mouse embryos overcame epigenetic barriers to embryo development at the 2-cell stage through elimination of H3K9 methylation of donor somatic cells; treatment of human embryos with the KDM4A isoform acted through a similar mechanism to overcome barriers at the 4- to 8-cell stage^14,15^. One histone modifying enzyme, coactivator-associated arginine methyltransferase 1 (CARM1), also known as protein arginine N-methyltransferase 4 (PRMT4), act in conjunction with other transcription factors, such as P53, NF_k_B, LEF1/TCF4 and TIF1a, to control gene expression^16–18^. CARM1 has also been shown to directly methylate histone 3 at arginine 2, 17 and 26 to control gene expression. Zernicka-Goetz and colleagues reported that endogenous CARM1 is involved in determining the fate of cells in 2-cell-stage embryos by inducing histone H3Arg26 (H3R26) dimethylation^13^. In embryonic stem cell (ESC) studies, CARM1 was shown to enhance the pluripotency of stem cells by increasing the expressions of *Oct4* (octamer-binding transcription factor 4), *Sox2* (SRY box 2), and *Nanog* (Nanog homeobox) genes^19^. In addition, increasing dimethylated H3R17 (H3R17me2) in human mesenchymal stem cells (MSCs) by treatment with CARM1 was shown to improve the expression of stemness-related genes and enhance differentiation-efficiency into mesodermal lineage cells^20^. However, the effect of treatment with exogenous CARM1 protein on pre- or post-implantation embryonic development has still not been well studied.

For epigenetic modification of an oocyte or embryo, mRNA, small interfering RNA (siRNA), or protein are typically injected into the cytoplasm through micromanipulation. However, this procedure is technically demanding and is likely to cause physical damage to the cloned embryo^14,15^. To avoid those limitations, many researchers have sought more facile and effective techniques for delivering verified factors into cells and embryos. It was recently reported that induced pluripotent cells (iPSCs) could be generated by introducing transcription factors fused with a cell-penetrating peptide (CPP)^21,22^. Lim and colleagues also identified a new form of CPP, obtained from human papillomavirus L1 capsid protein (LDP12), and confirmed its ability to deliver a fusion protein of enhanced green fluorescence protein and MAP1LC3 (EGFP-LC3) into mouse blastocysts^23^. In our previously reports, the cell number of inner cell mass (ICM) and expression of the *Oct4* gene were positively regulated in fresh and cryopreserved embryos after treatment with CPP-conjugated estrogen-related receptor β (CPP-ESRRB) during *in vitro* cultivation^24,25^. In the present study, we provide the first demonstration that exogenous supplementation with a novel CPP-conjugated CARM1 improves the normally poor embryonic development of cloned mouse embryos through regulation of gene expression.

## Results

### Construction of novel CPP-conjugated DsRed2 and CARM1 expression vectors

**Figure 1:**
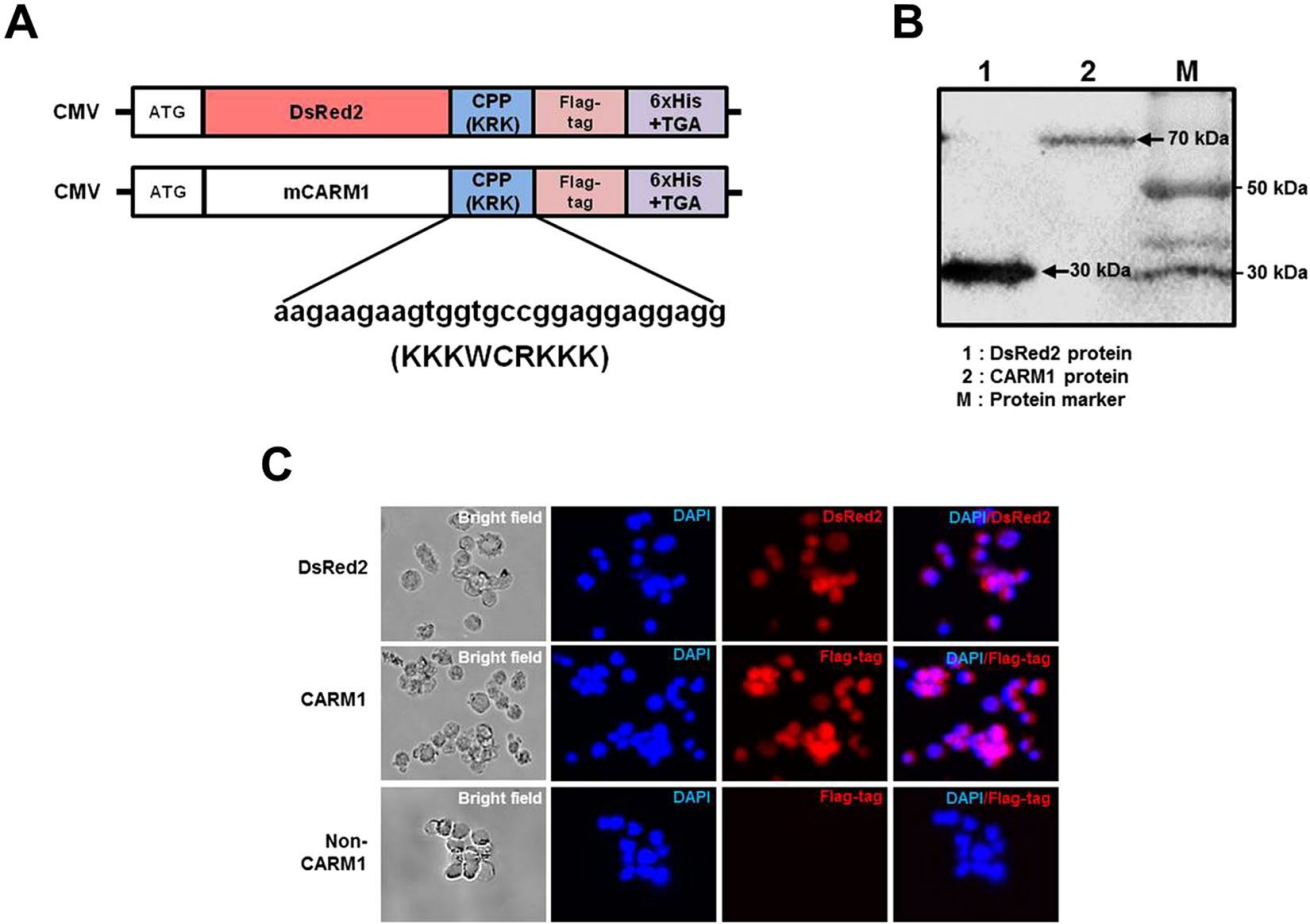
CPP-DsRed2 and CPP-CARM1 recombinant proteins. (**A**) Schematic diagrams of plasmid DNA expression constructs for recombinant proteins. CPP assists in the delivery of recombinant protein into target cells and hexa-histidine (6xHis) is required for purification of protein using anti-His agarose beads. ATG, start codon; DsRed2, red fluorescence protein; mCARM1, mouse coactivator-associated arginine methyltransferase 1; CPP, cell-penetrating peptide; 6x His + TGA: six-histidine sequence + stop codon. (**B**) DsRed2 and CARM1 protein expression were detected by Western blotting. Purified DsRed2 and CARM1 protein were resolved by SDS-PAGE and immunoblotted using an anti-FLAG antibody. CPP-DsRed2 and CPP-CARM1 proteins, including CPP peptide, protein tag (FLAG) and His_6_, were approximately 30 and 70 kDa, respectively. Lane M, protein marker; lane 1, CPP-DsRed2-FLAG protein; lane 2, CPP-CARM1-FLAG protein. (**C**) Analysis of DsRed2 and CARM1 protein localization and expression in mouse somatic cells by immunodetection of the FLAG tag and monitoring of red fluorescence, respectively.

Expression vectors designed to produce recombinant CPP-CARM1 and CPP-DsRed2 (internal control) proteins are shown in Fig. 1A. To enable efficient delivery of recombinant proteins, we fused a CPP (KRK) sequence to the C-terminus of each protein; purification was facilitated by incorporating a FLAG- and His_6_-tag at the C-terminal end of DsRed2- and CARM1-constructs (Fig. 1A). The purified recombinant proteins were confirmed by Western blot analysis using an anti-FLAG antibody. The size of CPP-DsRed2 and CPP-CARM1 proteins were approximately 30 and 70 kDa, respectively (Figs 1B and S1). The concentration of purified CPP-proteins was determined (Fig. S2) and adjusted to 20 µg/mL.

### Delivery of CPP-DsRed2 and CPP-CARM1 proteins into somatic cells and manipulated embryos

In a preliminary experiment, we examined the delivery of CPP-DsRed2 and CPP-CARM1 proteins into mouse cumulus cells (donor nuclei for nuclear transfer) and embryos by adding these proteins to culture medium at a 1:5 ratio (final concentration to 4 µg/mL). As shown in Fig. 1C, these proteins were first detected in all cumulus cells after 3 hours, but no morphological changes were observed in treated or untreated cells. We next analyzed the effect of CPP-conjugated protein treatment of nuclear donor cells (cumulus cells) on the development of SCNT mouse embryos. Cloned embryos obtained using CPP-CARM1-treated donor cells (CARM1-DSCNT group) showed an increase in 2-cell formation rate compared with that for CPP-DsRed2-treated donor cells (DsRed2-DSCNT group) (85.7 ± 1.3 *vs*. 77.8 ± 2.5, *p* < 0.05), but further development revealed no significant difference in blastocyst formation rate between the two groups (Table S2). In our preliminary study, we injected the *Carm1* mRNA into cloned mouse embryos after chemical activation (at 1-cell stage) and investigated their embryonic development. The developmental rate to 2-cell stage was increased in the *Carm1* mRNA-injected group compared to Sham injection group (*p* < 0.05), but blastocyst development of the *Carm1* mRNA-injected group was not different to that of the SCNT group and sham injection group, respectively (Table S3, *p* > 0.05). In addition, from the previously reports, overexpression of CARM1 in 4-cell embryo is involved in determination of the fate in embryonic blastomeres and of cell polarity in the development of preimplantation embryo^13,26^. So, in the present study, we have investigated the effect of CARM1-treatment on the cloned mouse embryonic development at the 8-cell stage (after decision of embryonic fate).

### Improved development of cloned embryos after supplementation with CPP-CARM1 protein

**Figure 2:**
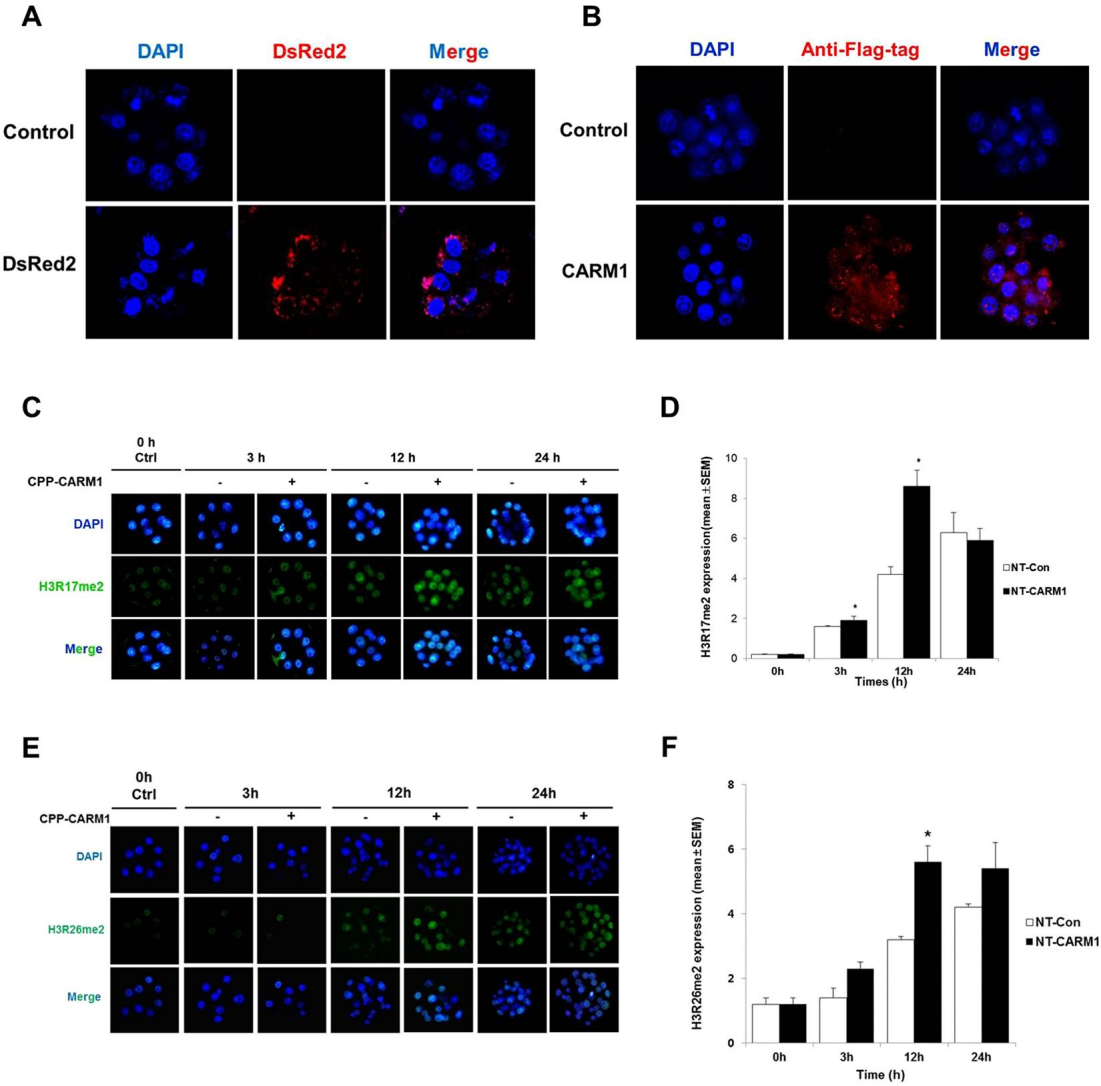
Analysis of H3R17me2 and H3R26me2 signals in mouse cloned embryos. (**A**,**B**) Analysis of DsRed2 and CARM1 protein localization and expression in mouse embryos by immunodetection of the FLAG tag and monitoring of red fluorescence, respectively. **(C**,**E**) Immunofluorescence staining of H3R17me2 and H3R26me2 in mouse cloned 8-cell embryos after treatment with recombinant CPP-CARM1 protein for 3 hours. Embryo samples were fixed at 0, 3, 12 and 24 hours. (**D**,**F**) H3R17me2 and H3R26me2 signal intensity was measured in mouse embryos after treatment with CPP-CARM1 protein at 0, 3, 12 and 24 hours. All of fluorescence image was exposed under the same condition (DAPI: 15sec-20sec and FITC: 300sec-350sec) using i-Solution program (i-Solution Inc, Burnaby BC Canada).The results are presented as means ± SEM. *p < 0.05.

To avoid changes of embryonic cell fate determined by endogenous CARM1 expression at 4-cell stage^13^, in the next experiment, we directly delivered CPP-conjugated proteins into mouse cloned 8-cell embryos and assessed changes in embryonic development. Exogenous DsRed2 was localized to the cytoplasm and CARM1 protein was detected primarily in the nucleus and to a lesser extent in the cytoplasm of all embryos after 3 hours (Fig. 2A,B). To analyze the functional effects of CPP-conjugated proteins, we investigated embryo morphology and histone methylation in treated embryos. Treatment with CPP-DsRed2 protein for 0, 3, 6 or 12 hours caused no adverse effects on embryonic development in any groups tested (Fig. S3A). In contrast, treatment with CPP-CARM1-protein for 3 hours caused much greater embryonic development compared with no treatment or treatment for 6 hours; however, treatment for 12 hours decreased blastocyst formation rate (Fig. S3B). On the basis of these results, we selected a 3-hour treatment for subsequent tests of CPP-CARM1 effects on embryogenesis.

After treatment of cloned 8-cell embryos with CPP-CARM1 for 3 hours, we analyzed specific arginine methylation signals and embryonic development. H3R17me2 signals were increased in embryos 3 and 12 hours after treatment with CPP-CARM1 compared with the untreated group, but, this difference was decreased 24 hours after treatment (Fig. 2C,D). As shown in Fig. 2E,F, H3R26me2 signals was significantly increased in embryos 12 hours after treatment with CPP-CARM1 compared to the untreated group. At 3 and 24 hours after CPP-CARM1 treatment, expression of H3R26me2 was increased but not significantly different from embryo untreated with CARM1. In addition, to investigate whether CARM1 is involved in other epigenetic modifications, we examined the expression patterns of DNA methylation which plays an important role in early embryonic development were analyzed. The DNMT3A showed hardly expression, while DNMT3B showed strong expression in the experimental groups (Fig. S3A,B). However, there was no difference in the expression of the two genes in the experimental group. Moreover, the expression pattern of H3K4me2, which is involved in gene transcription activity, and H3K9me3, which is involved in gene expression inhibition, were no difference in the NT-con group and the NT-CARM1-treated group (Fig. S3C,D). Therefore, this result suggested that CARM1 regulates gene expression by methylation the 17 and 26th arginine residues of Histone 3 without inducing any other epigenetic modification.

**Figure 3:**
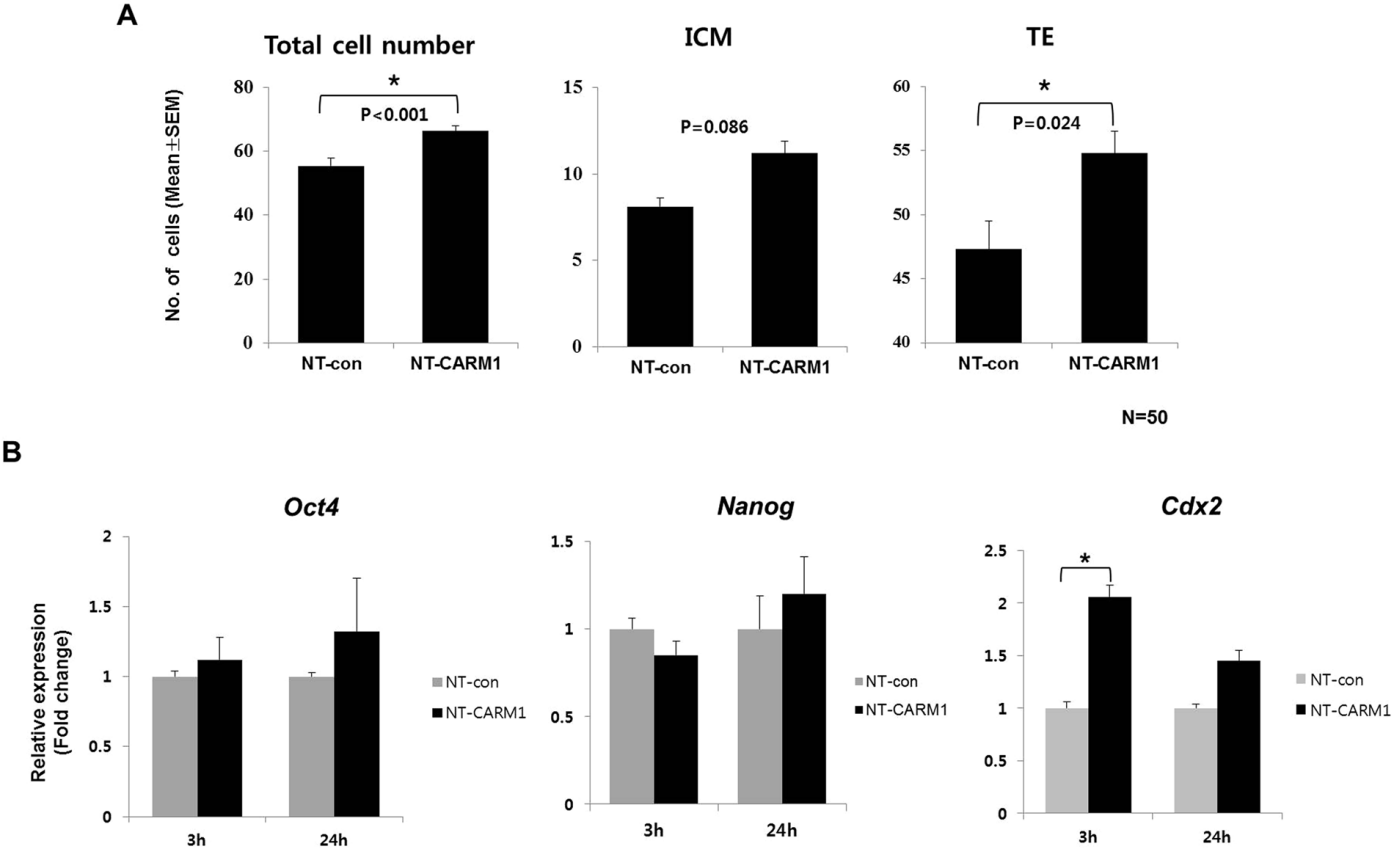
Effects of CPP-CARM1 protein on gene expression in mouse cloned embryos and embryo quality. (**A**) Analysis of total (TCN), ICM and TE cell numbers in blastocysts derived from NT-con and NT-CARM1 groups after CPP-CARM1 treatment at the 8-cell stage. (**B**) Relative expression levels of *Oct4*, *Nanog* and *Cdx2* in cloned embryos from NT-con and NT-CARM1 groups, determined by qRT-PCR. TCN, total cell number. Asterisks (*) indicate significantly different values (*p* < 0.05 and *p* < 0.001).

To analyze the effect of this histone methylation on embryonic development, we cultured protein-treated cloned embryos *in vitro* for two more days. At 120 hours post hCG, cloned embryos treated with CPP-CARM1 protein (NT-CARM1) showed a significantly higher blastocyst formation rate than CPP-DsRed2-treated embryos (NT-control) (Table 1, *p* < 0.05). Moreover, as shown in Fig. 3A, the numbers of total cells and trophectoderm (TE) cells in the NT-CARM1 group were significantly increased compared with the NT-control group (*p* < 0.05 and *p* < 0.001). The number of ICM cells in the NT-CARM1 group also trended higher, but this difference did not reach significance (Fig. 3A). These results suggest that CPP-CARM1 protein improves the development and quality of cloned embryos.

**Table 1:** Effect of CPP-CARM1 protein on the development of cloned 8-cell mouse embryos.

Groups	Number of embryos^*^ (Replication numbers, n)^**^	Number (Mean (%) ± SEM) of oocytes developed to the indicated stage		
		2-cell	8-cell	Blastocyst^***^
NT-control	306 (5)	275 (89.9)	119	68 (58.7 ± 2.5)^a^
NT-CARM1			117	83 (75.1 ± 5.9)^b^

^*^Somatic cell nuclear transferred (SCNT) embryos.

^**^The experiment was repeated five times for each group.

^***^Blastocysts were calculated from the number of 8-cell embryos.

^a,b^Within the same column, numbers with different superscripts are significantly different (*p* < 0.05); values in parentheses are mean (%) ± SEM.

### Regulation of TE-related gene expression and improvement of implantation rate

To investigate the effect of CARM1 on the specific regulation of blastocyst-related genes, we analyzed mRNA expression in NT-CARM1 and NT-control group embryos. The expression of *Oct4* and *Nanog*, marker genes for the ICM, did not significantly differ between the two groups at 3 and 24 hours after CPP-CARM1 treatment (Fig. 3B, *p* > 0.05). In contrast, expression of *Cdx2* (caudal type homeobox 2), a TE marker gene, was more highly expressed in the CPP-CARM1–treated group than in the control group (Fig. 3B, *p* < 0.05).

**Figure 4:**
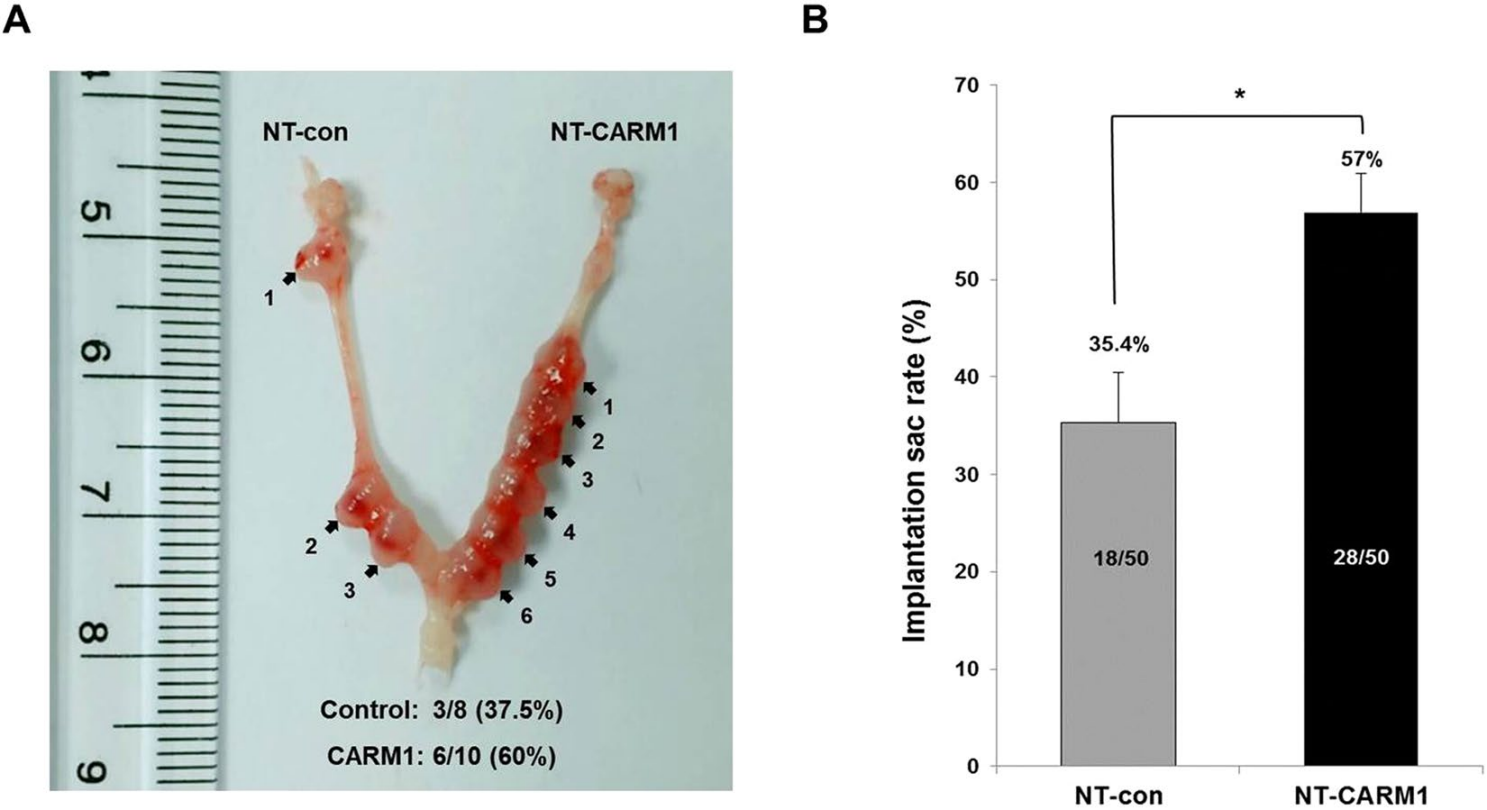
Analysis of implantation efficiency of cloned embryos treated with recombinant CPP-CARM1 protein. (**A**) Representation of the implantation sac and (**B**) diagram of implantation sac rate (%) for NT-control and NT-CARM1 groups. *p < 0.05.

To confirm effects on subsequent embryonic development, we transferred cloned embryos from each experimental group into uteri of recipients at the morula or early-blastocyst stage. At day 7.5 of embryo development, the implantation rate for NT-CARM1 group (57%; 28/50) was significantly higher than that for the NT-control (35.4%, 18/50) (Fig. 4A,B, *p* < 0.05). These results suggest that CPP-CARM1 protein enhances TE development of cloned embryos, resulting in an increased implantation rate.

## Discussion

Since the first production of the cloned sheep “Dolly” was reported^2^, cloned embryos from a variety of mammalian species have been successfully established by SCNT^3,4^. Cloned human embryos and embryonic stem cell lines have recently been produced by the SCNT technique using fetal^14^ and adult somatic cells^15^, suggesting that this technique may be applicable for the treatment of intractable diseases. However, cloning efficiency remains low. Among the various underlying causes is diminished embryonic development with arrested cell cycling or reduced cell numbers. These defects, which have been reported for most cloned embryos, reflect abnormal gene expressions attributable to abnormal DNA methylation and other epigenetic modifications. Therefore, rather than focusing on physical stimulation or genomic integration, in the present study, we investigated a method for up-regulating gene expression and increasing the development of cloned embryos using a protein fusion system for delivery system of histone methylase.

Since the initial introduction of virus-mediated production of iPSCs using Yamanaka factors (*Oct4*, *Sox2*, *Klf4*, and *c-Myc*)^27^, a number of different approaches have been tested for delivery of foreign genes into cells. However, the problem of genomic integration has remained a barrier to clinical application of virus-mediated gene delivery for cellular reprogramming^28^. In the current study, we chose KRK peptide from among several available cell-penetrating peptide sequences (e.g., 9R, 9K and novel peptides) for insertion into the expression vector used to produce recombinant proteins in mammalian cells. We confirmed the functionality of this CPP delivery system, showing that CPP-conjugated DsRed2 and CARM1 were incorporated into almost all somatic cells (Fig. 1C). However, we found that treatment of donor cells with CPP-CARM1 did not affect the blastocyst development of cloned embryos (Table S2). In fact, similar results were reported in previous studies that used donor cells treated with 5-aza-2′-deoxycytidine (AzC) for cloned bovine embryos^29^ and TSA or 5-AzC alone for cloning of minipig embryo^30^. Therefore, we concluded that treatment of donor cells with CPP-CARM1 protein was not suitable for the production of cloned mouse embryos. We thus subsequently applied recombinant CPP-CARM1 protein directly to cloned mouse embryos. Support for this strategy is provided by our previous report showing that CPP-conjugated ESRRB, a member of the orphan nuclear receptor families, improved the developmental potential of cryopreserved mouse embryos and increased expression of the transcription factors *Oct4* and *Nanog*^25^. In addition, supplementation of culture medium with CPP-ESRRB protein has been shown to enhance the function of ICM in fresh embryos increasing *Oct4* gene expression^24^. Consistent with these observations, we here found that treatment of embryos with CARM1 protein improved the TE development and enhanced the quality of cloned embryos through epigenetic modification.

It has been reported that CARM1 regulates cell fate and pluripotency in the mouse embryos through histone arginine methylation^13^. Notably, methylation of H3R26 sites by exogenously expressed CARM1 was found to induce the expression of *Sox2* and *Nanog* genes in blastomeres of the 4-cell embryo, which are destined to become the ICM of the blastocyst^13,26^. However, in this latter study, CARM1-mediated histone methylation after ICM determination and preimplantation embryo stages was not fully investigated. In the present study, treatment of cloned day 3 embryos with exogenous CARM1 increased total cell numbers in blastocysts by accelerating TE development. In fact, expression of *Cdx2* genes in cloned embryos was substantially increased after 3 hours of CPP-CARM1-treatment. Interestingly, after transfer of blastocysts into the uterus of the foster mother, the number of implantation sacs in the CPP-CARM1-treated group was substantially increased compared with that in the untreated group (Fig. 4). This suggests that up-regulation of H3R17me2 by treatment with CPP-CARM1 on day3 accelerates embryonic development by enhancing the proliferation of TE and improving the delayed development of cloned embryos. However, whether the histone modification system could be used to overcome the delayed development of *in vitro*-derived or cryopreserved embryos in conventional embryogenesis should also be examined.

In conclusion, we performed the first investigation to determine whether histone modification by exogenous treatment with recombinant CPP-CARM1 protein affects the development of cloned mouse embryos. Indeed, our novel CPP delivery system efficiently delivered CARM1 protein into somatic cells and embryos, and regulated embryonic gene expression and development of cloned embryo. These results suggest that induction of epigenetic modifications through delivery of CPP-conjugated CARM1 protein may be an effective method for regulating of embryogenesis and differentiation of stem cells *in vitro*.

## Methods

### Approvals of animal experiment

The protocol for the use of animals in these studies were approved by the Institutional Animal Care and Use Committee (IACUC) of CHA university (Approval number: IACUC-170056) and all experiments were carried out in accordance with the approved protocols.

### Animals and reagents

Adult (8–12 weeks) B6D2F1 (C57BL/6 J x DBA/2) and ICR mice were housed in ventilated racks and cages in a specific-pathogen-free (SPF) system at 20 °C. Mice were superovulated by injecting 5 IU of pregnant mare serum gonadotropin (PMSG; Sigma-Aldrich, St. Louis, MO, USA) and 5 IU human chorionic gonadotropin (hCG; Sigma-Aldrich), administered 48 hours apart. Mature oocytes were collected 14–15 hours after hCG injection. All reagents and chemicals were purchased from Sigma-Aldrich unless otherwise stated.

### Design of CPP (KRK)-conjugated CARM1 vector and purification of recombinant protein

Expression constructs for C-terminally FLAG- and His_6_-tagged recombinant proteins linked to the intracellular delivery sequence (KKKWCRKKK) and mCARM1 sequence^20^ were designed using the pcDNA3.1 vector. HEK 293 cells were maintained in high-glucose Dulbecco’s modified Eagle’s medium (DMEM; Invitrogen) containing 10% fetal bovine serum (FBS; Invitrogen), 2mM L-glutamine (Sigma-Aldrich), and 1% penicillin/streptomycin (Invitrogen). HEK 293 cells in 150 mm culture dish were transfected with 12.5 µg of CARM1 plasmid DNA using Lipofectamine 2000 (Invitrogen, Carlsbad, CA, USA). Two days after transfection, cells were washed with phosphate-buffered saline (PBS) and lysed with lysis buffer (50 mM NaH_2_PO_4_ (pH 8.0), 300 mM NaCl, 10 mM imidazole pH 7.0, 0.05% Tween-20, protein inhibitor cocktail, 1 mM PMSF) for 10 minutes on ice. Complete lysis was achieved by sonicating cells after which cells were centrifuged for 3 minutes at 3000 rpm. The protein-containing supernatant was incubated with Ni-NTA agarose bead (Qiagen, Hilden, Germany) for 1 hour, and bead-bound protein was eluted with elution buffer consisting of 50 mM NaH_2_PO_4_ (pH 8.0), 300 mM NaCl, and 250 mM Imidazole (pH 7.0). The imidazole concentration was then diluted by dialyzing the eluted protein against PBS. Protein concentration was determined using a BCA assay kit (Thermo-Fisher Scientific, Rockford, IL, USA) according to manufacturer’s instructions. The effects of CPP-conjugated proteins were analyzed by culturing donor cells for SCNT and cloned 8-cell embryos in media containing CPP-DsRed2 and CPP-CARM1, added at 1:5 ratio.

### Western blot analysis

Proteins in whole-cell lysates were resolved by sodium dodecyl sulfate-polyacrylamide gel electrophoresis (SDS-PAGE) on 12% gels, after which proteins were transferred to a polyvinylidene fluoride (PVDF) membrane (BIO-RAD, Hercules, CA, USA). After blocking for 1 hour with 5% skim milk, the membrane was incubated overnight at 4 °C with anti-FLAG antibody (Sigma-Aldrich), diluted 1:2000. It was then incubated for 1 hour with a biotin antibody (1:2000 dilution; Vector Laboratories, Burlingame, CA, USA), and then for 15 minutes with a streptavidin antibody (1:3000 dilution; Jackson ImmunoResearch Laboratories, West Grove, PA, USA). Immunoreactive proteins were detected using enhanced chemiluminescence (ECL) reagents (WelProtTMHRP Detection Kit, WelGENE, Daegu, Korea) and ChemiDoc machine (BIO-RAD).

### Somatic cell nuclear transfer

SCNT was carried out as described previously^15^ with slight modifications. Briefly, mature MII oocytes were collected from the oviducts of superovulated 8–12 week-old female mice. Oocytes were placed in 0.1% hyaluronidase until the cumulus cells dispersed. The oocytes were then placed in M2 medium containing HEPES (Sigma-Aldrich), and rinsed three times to remove the hyaluronidase solution. After oocyte collection, dispersed cumulus cells (nuclear donor for SCNT) were washed in M2 medium and then transferred to the SCNT dish in 12% polyvinylpyrrolidone (PVP, Sigma-Aldrich) drops until used.

Each forty to fifty oocytes were placed into a M2 medium supplement with 5 µg/mL cytochalasin B (CB, Sigma-Aldrich) for SCNT. The oocyte was rotated so as to place the spindle between the 8 and 10 o’clock position, and then was firmly attached to the holding pipette. The MII chromosome-spindle complex was removed by aspirating the enucleation pipette in conjunction with a piezo system (Prime Tech Ltd., Japan) without breaking the oolemma. The collected donor cell was resuspended in a drop of HVJ-E extract (Cosmo Bio, Japan) and then inserted into the perivitelline space of the enucleated oocyte. Fusion between the cytoplasm and cell membrane in reconstituted oocytes was confirmed after incubation for 15 minutes at 37 °C in air. The fused oocytes were placed in M16 medium (Millipore, Billerica, MA, USA) containing 5 µg/mL CB, 10 mM SrCl_2_ (strontium chloride), and 2 mM EDTA for 5 hours.

### *In vitro* transcription of mRNA and injection

*In vitro* transcription (IVT) was carried out described previously^15^. Full length of mouse Kdm4a and CARM1 cDNA was cloned into a pcDNA3.1 plasmid containing poly(A)83 plasmid. The mRNA was synthesized using a mMESSAGE mMACHINE T7 Ultra Kit (Life technologies # AM1345) following manufacturer’s instructions. The synthesized mRNA was dissolved in nuclease-freewater. The concentration of mRNA was analyzed by NanoDrop ND-1000 spectrophotometer (NanoDrop Technologies) and then aliquots were stored at –80 °C until use. The pronuclear stage of the cloned embryos were injected with ~10 pl of water (control), 1.9–2.0 µg/mL *Kdm4a* and *Carm1* mRNA at 5–6 hpa by using a Piezo-driven micromanipulator manipulator and then were cultured in KSOM medium (Millipore) for up to 5 days in a 5% CO_2_ incubator at 37 °C in air.

### Embryo transfer

Ten cloned morula or early-blastocyst stage embryos were transferred into the uteri of pseudopregnant females at 2.5 days post coitus (dpc). Implantation rate, calculated as the number of implanted sac per transferred embryos, was evaluated at 7.5 days after embryo transfer.

### Immunofluorescence staining

Immunofluorescence staining was as described previously^24^ with slight modifications. Briefly, the zona-pellucida of the embryo was removed using acidic Tyrode’s solution (Millipore). Zona-free embryos were rinsed three times with PBS (Hyclone, Logan, UT, USA) supplemented with 0.1% polyvinyl alcohol (PBS/PVA) and then fixed in 100% methanol (Millipore) at −20 °C for 10 minutes. Thereafter, embryos were permeabilized in PBS supplemented with 0.1% Tween-20 and 0.1% Triton X-100 at 4 °C for 15 minutes, and then blocked in PBS supplemented with 0.1% Tween-20 and 1% BSA (Sigma-Aldrich) for 1 hour. After blocking, embryos were incubated with anti-FLAG (1:100, rabbit polyclonal; Sigma-Aldrich), anti-H3R17m2 (1:100, rabbit polyclonal; Novus Biologicals, Littleton, CO, USA) and anti-OCT4 (1:100, mouse monoclonal, BD Biosciences) primary antibodies overnight at 4 °C. Thereafter, embryos were incubated in the dark for 1 hour at room temperature with Alexa Fluor 568-labeled goat anti-rabbit IgG or 488-labeled chicken-anti rabbit IgG (1:200 dilution; Molecular Probes, Eugene, OR, USA), as appropriate. Subsequently, embryos were placed in 1 μg/mL of 4′,6′-diamidino 2-phenyindiol (DAPI; Sigma-Aldrich) for 15 minutes, rinsed three times with PBS/PVA, placed on a glass slide, and coverslip mounted. The process for staining somatic cells was the same as that for embryos except the zona-pellucida elimination step was omitted. All sample images were captured using a confocal microscope (Zeiss LSM880) and an Olympus IX71 epifluorescence microscope controlled by ZEN 2012 (Zeiss) and DP manager version 3.1.1 software (Olympus). All images were analyzed using Image J (ver. 1.43).

### Quantitative reverse transcription-polymerase chain reaction

Total RNA was extracted from twenty 8-cell or morula-stage embryos in each experimental group using a manual process. In brief, embryos were transferred into 500 μL of TRIzol reagent (extraction buffer, Invitrogen) at −80 °C for 30 minutes. After lysis of embryos, 100 μL of chloroform (Sigma-Aldrich) was added and the solution was allowed to stand at room temperature for 3 minutes. After centrifuging the solution at 14,000 rpm for 15 minutes, the supernatant was transferred to a new sterile microfuge tube, and 1 μg of glycogen (Sigma-Aldrich) was added. Following, 20 μL of 3 M sodium acetate and 221 μL of iso-propanol were added and the solution was kept at −80 °C overnight. The next day, the supernatant was removed by centrifugation at 14,000 rpm for 10 minutes at 4 °C, 1000 μL of 75% ethanol was added to the pellet and the tube was centrifuged at 14,000 rpm for 5 minutes at 4 °C. The ethanol was subsequently removed by heating in a dry oven at 37 °C for 10 minutes, and the precipitated total RNA was dissolved in 20 μL of sterile water. cDNA was synthesized from 1 μg of total RNA using a TOPscript cDNA synthesis Kit (Enzynomics, Seoul, Korea) according to the manufacturer’s instructions. Quantitative reverse transcription-polymerase chain reaction (qRT-PCR) analyses of cDNA samples were performed using a CFX98 instrument (Bio-Rad) according to a previously published report^24^ with slight modifications. Briefly, all cDNA samples were analyzed by qRT-PCR using primers designed to amplify H2A histone family member Z (H2afz) to test for variations in the expression of this internal control gene. The thermocycling conditions used for quantifying genes of interest were 15 minutes at 95 °C, then 46 cycles of 10 seconds at 95 °C, 30 seconds at 60 °C and 30 seconds at 72 °C, followed by a final 60-second extension at 60 °C (Table S1). Results were analyzed using the 2^-△△^Ct method, and expression levels were reported relative to that of the calibrator after normalization of the target transcript to the endogenous control.

### Statistical analysis

Embryo development, cell number, H3R17me2 expression and relative gene expression values are expressed as means ± standard error (SEM). Implantation rate is presented as a percentage. All experiments were repeated at least three times. Experimental data were analyzed using Student’s *t-*test and chi-square test in the Statistical Package for the Social Sciences (SPSS, ver. 18; SPSS Inc., Chicago, IL, USA). *P*-values < 0.05 were considered statistically significant; individual *p*-values are presented in figure legends.

## Electronic supplementary material

SUPPLEMENTARY INFORMATION
