# Supplementary material for: The effect of cell penetrating peptide-conjugated coactivator-associated arginine methyltransferase 1 (CPP-CARM1) on the cloned mouse embryonic development
Source: Sci Rep. 2018 Nov 13;8:16721. doi: 10.1038/s41598-018-35077-0 (PMC6233168; doi:10.1038/s41598-018-35077-0)
Supplement: Supplementary file 1 — SUPPLEMENTARY INFORMATION [file 41598_2018_35077_MOESM1_ESM.docx]

SUPPLEMENTARY INFORMATION

**Title: The effect of cell penetrating peptide-conjugated coactivator-associated arginine methyltransferase 1 (CPP-CARM1) on the cloned mouse embryonic development**

**Authors:**

**Jae-Il Bang^a,1^, Eun-Hye Lee^b,1^, Ah Reum Lee^a^, Jin Il Lee^c^, Seo Hye Choi^a^, Dong-Won Seol^a^, Chang-Hwan Park^b,*^, and Dong Ryul Lee^a,d,^*^*^***

^a^ Department of Biomedical Science, CHA University, Seongnam 13488, Korea,

^b^ Graduate School of Biomedical Science and Engineering, Hanyang University, Seoul 04763, Korea,

^c^ Fertility Center of CHA Gangnam Medical Center, College of Medicine, CHA University, Seoul 06135 Korea.

^d^ CHA Stem Cell Institute, CHA University, Seongnam 13488, Korea

^1^ These authors contributed equally to this study.

**Running Head:** CPP-CARM1 regulates the development of cloned embryos

***^*^ To whom co-correspondence may be addressed:*** **Dong Ryul Lee, Ph.D.,** Department of Biomedical Science, College of Life Science, CHA University, 335 Pankyo-ro, Seongnam-si, Gyeonggi-do 13488, Korea. Tel: +82-31-881-7136, E-mail: [drleedr@cha.ac.kr](mailto:drleedr@cha.ac.kr); **Chang-Hwan Park, M.D., Ph.D.,** Graduate School of Biomedical Science and Engineering, Hanyang University, Seoul 04763, Korea, Tel: +82-2-2220-0646, E-mail: [chshpark@hanyang.ac.kr](mailto:chshpark@hanyang.ac.kr)

**Supplementary Table 1.** Primers for qRT-PCR

| **Gene name** |  | **Sequences** | **Size** |
| --- | --- | --- | --- |
| *Oct4* | F: | 5’-CTGAGGGCCAGGCAGGAGCACGAG-3’ | 485bp |
|  | R: | 5’-CTGTAGGGAGGGCTTCGGGCACTT-3’ |  |
| *Nanog* | F: | 5’-AGGGTCTGCTACTGAGATGCTCTG-3’ | 364bp |
|  | R: | 5’-CAACCACTGGTTTTTCTGCCACCG-3’ |  |
| *Cdx2* | F: | 5’-CTACGGCGAACTTGGACA-3’ | 200bp |
|  | R: | 5’-GTGATGGTGCGCGTGGTA-3’ |  |
| *H2afz* | F: | 5’-ACAGCGCAGCCATCCTGGAGTA-3’ | 202bp |
|  | R: | 5’-TCCCCGATCAGCGATTTGTGGA-3’ |  |

**Supplementary Table 2.** Developmental potential of cloned embryos derived from donor cells treated with CPP-DsRed2 (control) or CPP-CARM1 protein

| Group | Donor cell | Number of embryos*  (Replication number, n)** | Number (Mean (%) ± SEM) of embryos developed to the indicated stage | |
| --- | --- | --- | --- | --- |
|  |  |  | 2-cell | Blastocysts*** |
| NT-DsRed2 (control) | CCs^§^ | 106 (4) | 83 (77.8±2.5)^a^ | 29 (34.6±7.9)^a^ |
| NT-CARM1 |  | 105 (4) | 90 (85.7±1.3)^b^ | 24 (26.9±5.0)^a^ |

^§^CC: cumulus cells

* Somatic cell nuclear transferred (SCNT) embryos

** The experiment was repeated designated times for each group.

*** Blastocysts were calculated from 2-cell embryos.

^a,b^ Within the same column, numbers with different superscripts are significantly different (*p* < 0.05); values in parentheses are mean (%) ± SEM.

**Supplementary Table 3.** Developmental potential of cloned embryos derived from *Carm1* mRNA-injected SCNT embryos at the pronuclear (PN) stage

| Groups | Concentration of mRNA (μM) ^§^ | Number of embryos^*^  (Replication number, n)^**^ | | Number (Mean (%) ± SEM) of embryos developed to the indicated stage | | |
| --- | --- | --- | --- | --- | --- | --- |
|  |  |  |  | PN | 2-cell | Blastocysts******* |
| Sham injection | - | 52(2) | 44(84.2±5.1) | | 21(48.3±4.3)^a^ | 6(28.6±1.4)^a^ |
| SCNT | - | 89(5) | 86(97±1.9) | | 67(79.7±5.9)^b^ | 19(28.6±3.2)^a^ |
| SCNT-*Carm1*  *mRNA*  Injection | 2.0 | 100(4) | 95(94.5±1.7) | | 71(74.3±10.2)^b^ | 11(14.5±5.7)^a^ |

^§^ *Carm1* mRNA was transcribed *in vitro*

* Somatic cell nuclear transferred (SCNT) embryos

** The experiment was repeated designated times for each group.

*** Blastocysts were calculated from 2-cell embryos..

^a,b^ Within the same column, numbers with different superscripts are significantly different (*p* < 0.05); values in parentheses are mean (%) ± SEM.


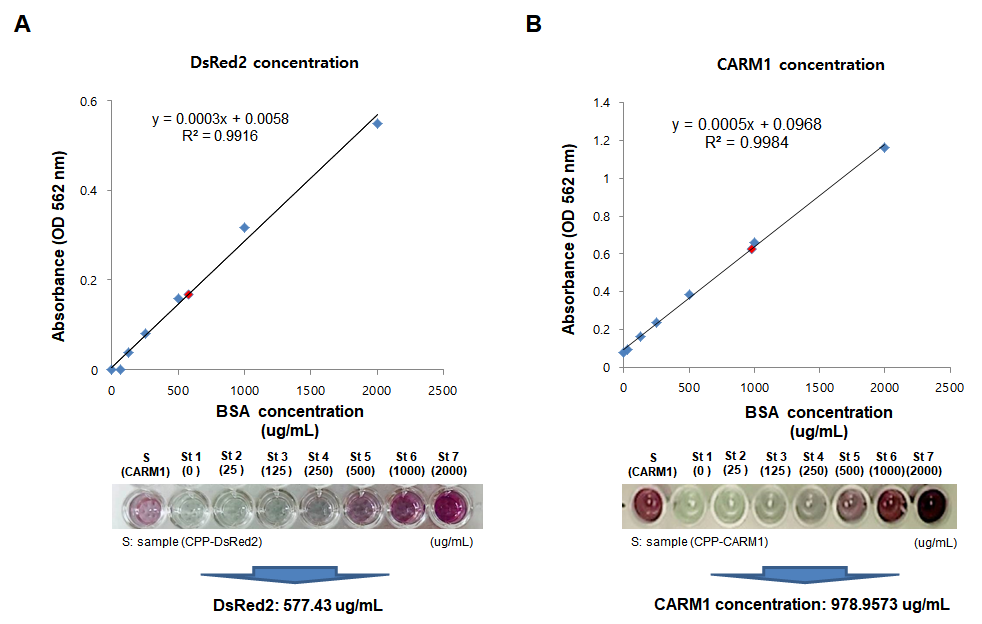


**Supplementary Figure 1. Concentrations of recombinant CPP-DsRed2 and CPP-CARM1 protein.** (A and B) Graph of DsRed2 and CARM1 protein concentration (A) and photograph of the microplate (B). Protein concentration determined by measuring absorbance at 562 mm. S, protein samples (DsRed2 and CARM1); st1–st7, BSA standard samples (0, 25, 125, 250, 500, 1000 and 2000 µg/mL).

**
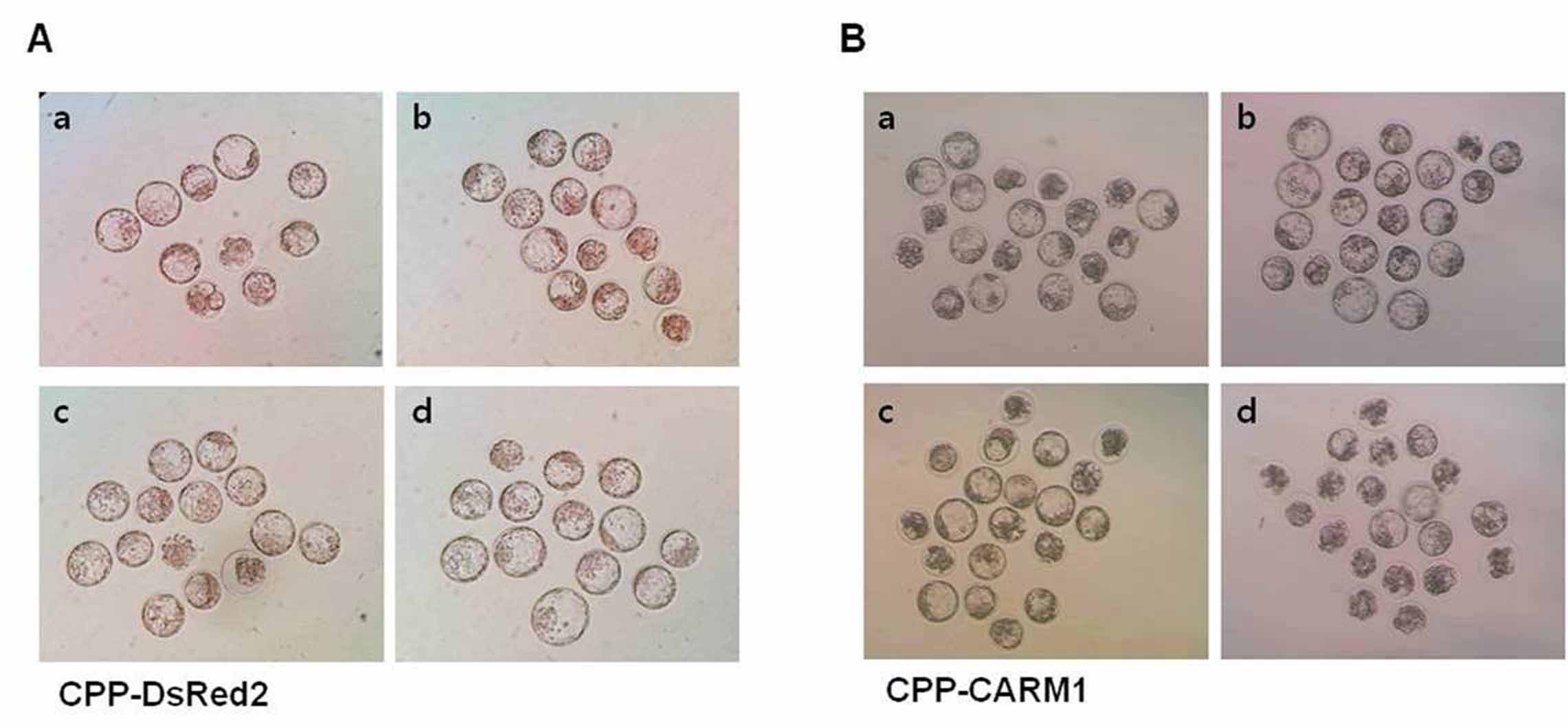
**

**Supplementary Figure 2. Effects of DsRed2 and CARM1 protein treatment duration on embryonic development up to the blastocyst stage.** Representative examples of embryonic development (blastocyst images) from CPP-DsRed2 (A) and CPP-CARM1 (B) groups treated for 0 hours (a), 3 hours (b), 6 hours (c) or 12 hours (d).

**
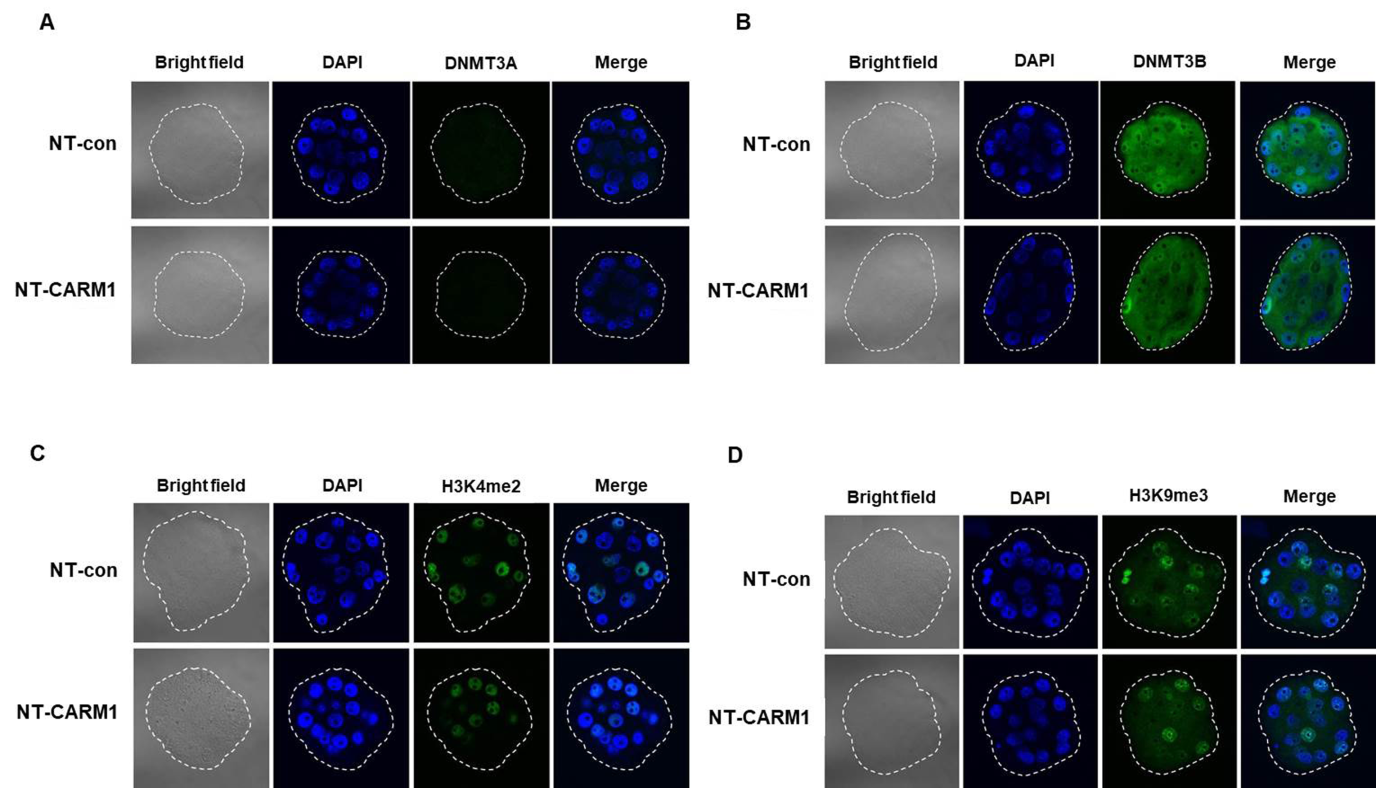
**

**Supplementary Figure 3. Analysis of epigenetic gene signals in mouse cloned embryos after CPP-CARM1 treatment.** Immunofluorescence stained embryos with anti-DNMT3A antibody (A), anti-DNMT3B antibody (B), anti-H3K4me2 antibody (C) and anti-H3K9me3 antibody (D). The nuclei were labeled with 4’,6’-diamidino-2-phenylindole (DAPI). Each group of embryo shape was indicated using a white dotted line.
